# From histology to spatial transcriptomics: establishing a lightweight single-patch baseline

**DOI:** 10.1186/s12859-026-06447-7

**Published:** 2026-05-18

**Authors:** Hyungyum Jang, Hyunsoo Shin, Hawon Lee, Yena Jang, Sung Hoon Jung, Minji Jeon

**Affiliations:** 1 Department of Applied Artificial Intelligence, Hansung University, Samseongyo-ro 16-gil 116, Seongbuk-gu, Seoul, 02876 South Korea; 2 Department of Applied IT Engineering, Hansung University College of IT Engineering, Samseongyo-ro 16-gil 116, Seongbuk-gu, Seoul, 02876 South Korea; 3 Department of Biomedical Sciences, Korea University College of Medicine, Jeongneung-ro 161, Seongbuk-gu, Seoul, 02708 South Korea; 4 Inje University College of Medicine, 75 Bokji-ro, Busanjin-gu, Busan, 47392 South Korea; 5 Department of Biomedical Informatics, Korea University College of Medicine, Jeongneung-ro 161, Seongbuk-gu, Seoul, 02708 South Korea; 6 Department of Electrical and Electronic Engineering, Yonsei University, 50 Yonsei-ro, Seodaemun-gu, Seoul, Seoul, 03722 South Korea; 7 Department of Applied Artificial Intelligence, Hansung University, Samseongyo-ro 16-gil 116, Seongbuk-gu, Seoul, 02876 South Korea

**Keywords:** Spatial transcriptomics, Computational pathology, H& E histology, EfficientNet, Deep learning, Bioinformatics

## Abstract

Predicting spatial gene expression from hematoxylin and eosin (H&E)-stained histological images is a central challenge in the field of computational pathology. Although many models leverage the spatial context from entire whole-slide images to boost performance, the predictive capability achievable from single, isolated tissue patches remains unexplored. Without this fundamental baseline, it is difficult to fairly measure the marginal benefit of incorporating spatial context. In this study, we established a lightweight, efficient, and reproducible baseline for single-patch gene expression prediction, thereby providing a standardized foundation for future research. We trained various convolution-based pretrained architectures, such as EfficientNet, ResNet, and DenseNet, using a fixed data split and seed on a curated human liver Visium dataset. To assess cross-tissue generalizability, we further validated the proposed baseline on an independent breast cancer spatial transcriptomics dataset. Through full fine-tuning and morphology-preserving augmentation, EfficientNet-B0 achieved a maximum Pearson correlation coefficient (PCC) of 0.310 for highly expressed genes on liver tissue, surpassing previous single-patch methods. Furthermore, the model predicted 50 genes with PCC *\ge * 0.30 using only 5.3M parameters-−2.5*× * more genes than a ResNet-50-based baseline despite using 5*× * fewer parameters–suggesting a favorable performance-efficiency tradeoff within this single-patch setting. On the independent breast cancer dataset, EfficientNet-B0 consistently achieved best or near-best performance across all gene sets under both single-donor and multi-donor evaluations, demonstrating stable generalization across tissue types. Subsequent biological analysis reveals that model prediction accuracy is primarily driven by the strength of spatial organization in H&E histology, measured by Moran’s I spatial autocorrelation, rather than transcript abundance or chemical properties. Consequently, this work provides a reference for quantifying the contributions of spatial context, multiresolution integration, and architectural complexity in future spatial transcriptomics models.

**Supplementary Information:**

The online version contains supplementary material available at 10.1186/s12859-026-06447-7.

## Background

Spatial transcriptomics (ST) technologies have transformed molecular biology by enabling gene expression profiling within an intact tissue architecture. Through a combination of histological imaging and spatially resolved RNA sequencing, ST provides direct spatial maps of transcriptional activity, revealing cell–cell interactions and microenvironmental structures that bulk or single-cell RNA sequencing cannot capture [1, 2]. However, the practical implementation of ST remains challenging. The technique requires specialized instrumentation, delicate sample preparation, and intensive computational processing, leading to high costs and limited scalability compared with conventional histopathology [3, 4]. In contrast, hematoxylin and eosin (H&E)-stained slides are available in vast numbers in routine pathology workflows [5]. These slides capture cellular morphology at subcellular resolution, providing an easily accessible and inexpensive source of information regarding tissue architecture. If gene expression profiles could be reliably inferred from such images, this would enable large-scale molecular analysis using existing histopathological archives, overcoming the cost and accessibility barriers of current spatial transcriptomics technologies. This potential has driven a wave of computational efforts to predict spatial gene expression directly from H&E-stained tissue images using deep learning techniques.

Recent advances in computational pathology have demonstrated that the morphological features extracted from histology can encode biologically meaningful signals associated with gene expression and molecular pathways. Existing image-to-transcriptomic approaches can be broadly categorized into three methodological paradigms. The first is the foundation model approach, where large-scale self-supervised models such as Prov-GigaPath [6], UNI [7], and Virchow [8] are trained on millions of whole-slide images (WSIs) to learn generic morphological representations transferable to downstream tasks. Some multi-modal approaches, such as OmiCLIP, couple histology with transcriptomic data to align visual and molecular embeddings, thereby enabling cross-modal predictions and molecular interpretations [9]. The second paradigm involves multiresolution-based tissue-level contextual modeling, which captures global context and integrates features across multiple resolutions to exploit spatial continuity within the tissue. Recent examples include SEQUOIA, a multiresolution deep learning framework that uses linearized attention to integrate fine- and coarse-scale histological features, enabling efficient whole-slide inference and accurate digital reconstruction of spatial gene expression directly from tissue images without sequencing [10]; HGGEP, which introduces hypergraph neural networks to encode higher-order spatial relations among histological regions [11]; and TRIPLEX, which fuses information from the target patch, its local neighborhood, and broader whole-slide environment [12]. The third paradigm is a patch-level modeling approach that treats each ST patch as an independent unit and directly predicts its expression profile from the corresponding image patch. Early efforts such as ST-Net [13] and HisToGene [14] laid the foundation for this approach. Specifically, ST-Net utilizes a densely connected convolutional network, DenseNet-121 [15], to regress the gene expression levels directly from local image patches. By contrast, HisToGene employs a Vision Transformer architecture [16] to model spatial gene expression, leveraging self-attention mechanisms to capture contextual dependencies between tissue patches. Recently, BLEEP advanced this field by introducing a contrastive learning framework that aligns histological patches with gene expression embeddings, thereby improving the consistency of image-to-transcript mapping [17].

While foundation and multi-resolution approaches often achieve the highest overall accuracy, they introduce a fundamental ambiguity: their superior performance may arise not from improved patch-level learning but from the inclusion of a broader spatial context. Consequently, the field currently lacks a clear understanding of the extent to which molecular information can be extracted from a single, isolated H&E patch. Without a rigorously defined and reproducible patch-level baseline, it is difficult to quantify the true marginal benefit of adding spatial context, multiresolution fusion, or increased architectural complexity. Moreover, inconsistencies in data preprocessing, model initialization, and random seed selection across studies have further confounded fair comparisons, leading to uncertainty in the assessment of progress in the field. Crucially, without an isolated patch-level control, it remains unclear whether performance gains in complex models stem from superior feature extraction or simply from capturing broader spatial correlations. Therefore, it is critical to establish a standardized, reproducible, and computationally efficient baseline for single-patch gene expression prediction. This baseline serves two purposes. Methodologically, it defines the performance achievable from local morphology alone, without spatial context. This serves as a control for quantifying how much broader spatial information contributes to prediction accuracy. Practically, it offers a lightweight and accessible framework suitable for laboratories or institutions without extensive GPU resources or a large-scale digital pathology infrastructure. Thus, a patch-level benchmark facilitates fair comparisons among emerging models and promotes reproducibility in ST research. Despite the need for such a baseline, few studies have systematically examined model architectures, data-augmentation strategies, and fine-tuning protocols in a single-patch setting. Previous studies have often focused on high-capacity models such as ResNet-50 [18] or Vision Transformers [16], which, while powerful, require substantial computational resources and training times [19]. In addition, data-augmentation strategies in previous studies have often been optimized for natural images or whole-slide classification rather than for preserving histological morphology, which increases the loss of biologically meaningful features. These gaps underscore the need for a principled evaluation of model efficiency, generalization, and robustness under controlled experimental conditions.

To address these gaps, we aimed to establish a lightweight and reproducible baseline to predict single-patch spatial gene expression. Specifically, we systematically benchmarked multiple pretrained convolutional backbones under consistent data splits and training configurations using publicly available human liver and breast cancer spatial transcriptomics datasets. We applied an augmentation pipeline designed to improve robustness while maintaining tissue integrity and conducted a hyperparameter search to systematically optimize the backbone architecture, network depth, and fine-tuning scope under a unified framework. Furthermore, we conducted biological pathway analysis and statistical modeling to determine which gene characteristics drive predictability. This study provides a reproducible foundation for evaluating future models by benchmarking computationally efficient baseline models. The established framework allows for the objective assessment of spatial context, multiresolution design, and multimodal integration while promoting reproducibility and fairness in the benchmarking of ST models.

## Methods

### Dataset preparation and preprocessing

**Fig. 1:**
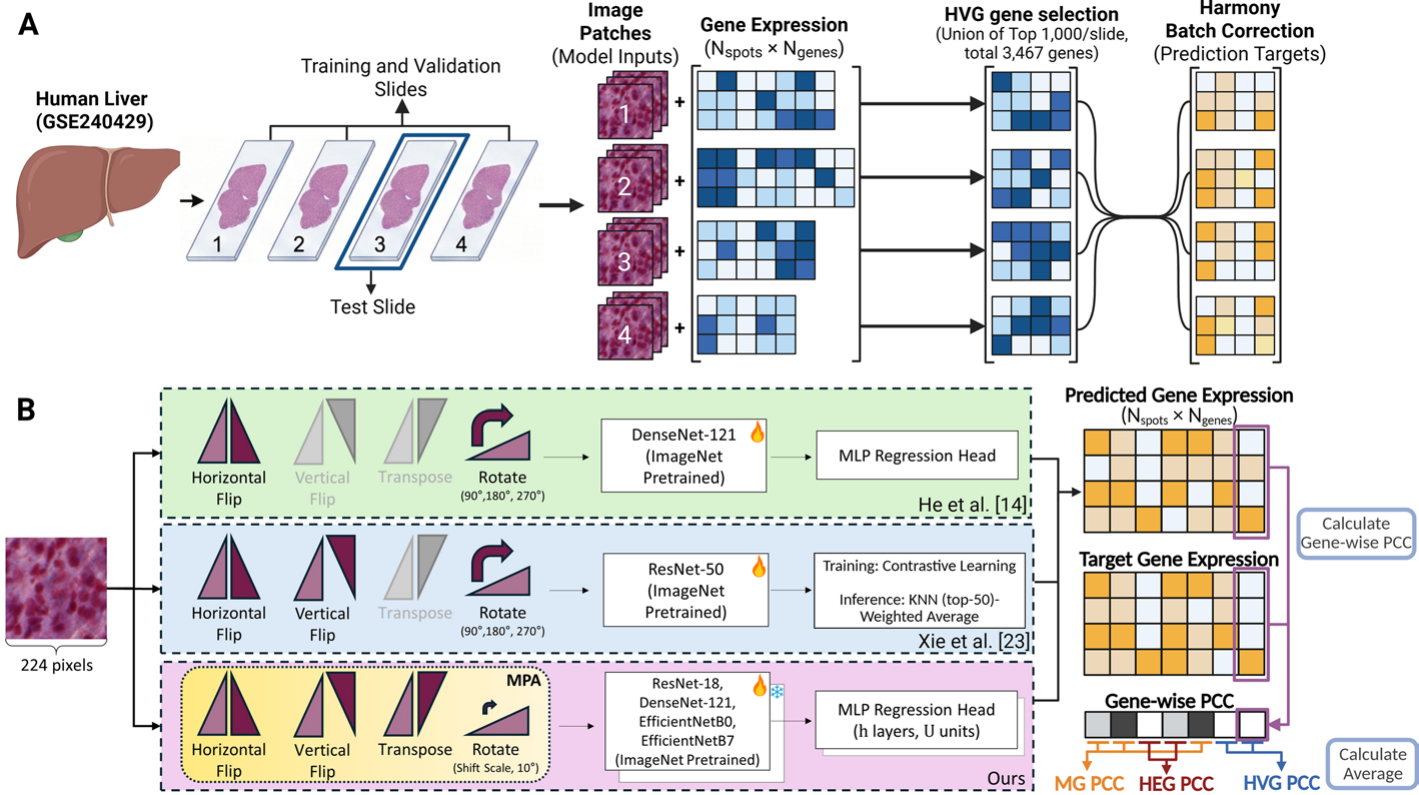
Overview of dataset preparation and evaluation pipelines. **A** Slide-level data split for the human liver Visium dataset (GSE240429), image–expression pair extraction, HVG gene selection (union of top 1,000 per slide, yielding 3,467 genes), and Harmony batch correction. **B** Comparison of ST-Net [13], BLEEP [17], and the proposed benchmark framework for patch-level gene expression prediction. The frameworks differ in augmentation strategy, backbone architecture, learning scheme, and prediction head, and are evaluated using gene-wise PCC over MGs, HEGs, and HVGs

To establish a reproducible performance baseline, we used a publicly available ST dataset: the de-identified human liver Visium dataset (GSE240429) [20]. This dataset comprises four consecutive liver sections obtained from a single healthy human liver and profiled on the 10x Genomics Visium platform. Each section was obtained from a $16\,\mu \textrm{m}$ frozen slice stained with H&E and includes paired H&E patch images and spatial gene expression values for approximately 2,300 capture spots per slide. For each spot, we extracted a *224× 224*-pixel image patch centered on the spot, corresponding to an area of approximately $55 \,\mu \textrm{m} \times 55\,\mu \textrm{m}$, and paired it with the corresponding expression vector. Across the four slides, this process yielded 9,269 image–expression pairs. We clarify that these pairs represent the aggregate of local image–expression instances used for model training and evaluation, rather than independent biological replicates. An overview of the dataset split and preprocessing pipeline is illustrated in Fig. 1A.

To ensure consistency with prior studies, we followed a preprocessing workflow used in BLEEP [17]. For each spot, raw gene expression counts were normalized to the total count using Scanpy [21], followed by natural-log transformation. For each slide, the top 1,000 highly variable genes were identified, and the union across the four slides yielded 3,467 unique prediction targets. The expression matrices were then batch corrected using Harmony [22] to mitigate inter-slide technical variation.

To evaluate model prediction performance while preventing information leakage from spatially adjacent patches, we employed a slide-level data split. One slide (slide 3, containing approximately 2,300 spots) was used as the test set, and the remaining slides were used for training and validation. By following the split protocol established in BLEEP [17], we adopted a slide-level data split to evaluate baseline model performance on unseen slides. This ensures that neighboring patches from the same tissue section do not appear in both the training and test sets, while also enabling direct comparison with prior benchmark results.

To evaluate generalizability across tissue types, we used an independent breast cancer spatial transcriptomics dataset [13, 23]. Four patients (BC23803, BC24105, BC24220, BC23270) were randomly selected, each containing three consecutive tissue sections. Following the liver dataset preprocessing protocol, raw gene expression counts were normalized, log-transformed, and subjected to Harmony batch correction to remove patient-specific technical variation. We used 60 genes, including 50 highly expressed genes (HEGs), 50 highly variable genes (HVGs), and 8 established breast cancer marker genes (MG) as prediction targets. To examine the effect of the split strategy on performance, we adopted two settings: a slide-level split within individual patients and a patient-level split across patients. For all slide-level experiments, slide IDs within each patient were sorted in alphanumeric order and assigned sequentially to training, validation, and test sets. For the patient-level evaluation, patients were randomly assigned to training (BC23803, BC24105), validation (BC24220), and test (BC23270) sets, which prevents any data leakage between samples from the same patient.

### Data augmentation: morphology-preserving augmentation

Data augmentation is essential for improving model generalization in histopathological image analysis. This helps the model become invariant to technical artifacts arising from staining variability, tissue preparation, and imaging conditions, thereby allowing it to focus on biologically meaningful morphological features. Previous studies, such as ST-Net [13], employed simple transformations, including random vertical flips and $90^{\circ }$ rotations. While these rigid transformations inherently preserve tissue structure, they are insufficient for simulating diverse geometric variations and staining inconsistencies arising from tissue preparation and imaging conditions.

To address this limitation while rigorously maintaining histological integrity, we formalized a Morphology-Preserving Augmentation (MPA) pipeline using the Albumentations library [24]. MPA consists of four operations: HorizontalFlip, VerticalFlip, Transpose, and ShiftScaleRotate. Formally, let $I(\textbf{x})$ denote an input image patch, where $\textbf{x} = (x, y)^T$ represents pixel coordinates. MPA applies transformations sequentially:

1 $$\begin{aligned} & \textbf{x}' = \textbf{A}(s,\theta ,\textbf{t}) \circ \textbf{F}(\textbf{x}), \end{aligned}$$

2 $$\begin{aligned} & \textbf{A}(s,\theta ,\textbf{t})= \begin{bmatrix} s \cos \theta & -s \sin \theta & t_x \\ s \sin \theta & s \cos \theta & t_y \\ 0 & 0 & 1 \end{bmatrix}, \qquad \textbf{F}= \begin{bmatrix} \textbf{F}_{2\times 2} & \textbf{0}\\ \textbf{0}^\top & 1 \end{bmatrix}. \end{aligned}$$

Here, $\textbf{F}$ denotes reflection operations (HorizontalFlip, VerticalFlip, and Transpose), and $\textbf{A}$ represents the shift-scale-rotate transformation that applies scaling $s \sim \mathcal {U}(0.9, 1.1)$, rotation $\theta \in [-30^\circ , 30^\circ ]$, and translation $(t_x/W, t_y/H) \sim \mathcal {U}(-0.1, 0.1)$, each with probability *p=0.5*. The matrix $\textbf{F}_{2\times 2}$ captures the composition of reflection operations, each applied independently with *p=0.5*.

These transformations preserve angles and relative distances, thereby maintaining cellular morphology and tissue architecture. Aggressive augmentations, including cutout and elastic deformation, were excluded to prevent distortion of biologically meaningful histological structures.

### Training protocol and evaluation metrics

To establish a reproducible and fair baseline, we trained multiple pretrained convolutional architectures under a unified experimental setting. Specifically, we evaluated ResNet-18 [18], DenseNet-121 [15], EfficientNet-B0, and EfficientNet-B7 [25] and compared their efficiency and predictive performance in spatial gene expression inference.

#### Baseline configuration

All the architectures followed a shared baseline configuration to ensure comparability. Each *224× 224* H&E patch was processed using a pretrained backbone to extract feature embeddings. These embeddings were then passed to a multi-output regression head implemented as a multi-layer perceptron (MLP) with *h* hidden layers and *U* units to predict 3,467 gene expression values. The specific dimensions for *U* and *h* were identified through the optimization strategy described below. The overall framework is shown in Fig. 1B.

#### Hyperparameter search

Hyperparameter optimization was performed via 5-fold cross-validation using all patches from the three training slides. To efficiently explore the large hyperparameter space, we adopted a hierarchical coarse-to-fine optimization strategy using mean squared error (MSE) and Pearson correlation coefficient (PCC).

***Stage 1: Architecture Selection*** This stage identified the optimal backbone architecture and hidden layer size *U*. We tested four backbones–ResNet-18, DenseNet-121, EfficientNet-B0, and EfficientNet-B7–with $U \in \{1024, 3000, 4096\}$ and depths $h \in \{1, 2\}$ for finer comparison. Evaluation was based on validation loss and consistency across MGs, HEGs, and HVGs. The top-performing backbone family and most stable hidden layer size were selected for stage 2.

***Stage 2: Fine-grained Optimization*** Focusing on the screened backbone family and optimal hidden layer size from stage 1, we optimized network depth $h \in \{0, 1, 2\}$, where *h=0* denotes a linear baseline, and fine-tuning scope $f \in \{0, \dots , 6\}$, which specifies the number of frozen backbone layers. We selected the configuration achieving the highest validation PCC. Using this optimal configuration, three independent training runs were conducted with different random seeds, and their test set performance was evaluated.

#### Evaluation metrics

For each target gene, we computed the PCC between the predicted and observed expression values across all spots in the held-out test set. The resulting gene-wise PCC values were then grouped into three gene sets (MGs, HEGs, and HVGs) and averaged within each set, with results summarized over three independent random seeds. We report the mean across seeds together with the maximum deviation from that mean to quantify variability. Performance was evaluated on three biologically relevant gene sets: (1) MGs, eight liver marker genes from Andrews et al. [30]; (2) HEGs, the 50 genes with the highest mean expression across spots; and (3) HVGs, the 50 genes with the greatest residual variance. These complementary sets captured the distinct biological expression patterns.

#### Implementation details

All models were trained using MSE loss and optimized using AdamW. The initial learning rate was set to $3\times 10^{-4}$ with a cosine annealing schedule. Each model was trained for up to 30 epochs with a batch size of 32 under mixed-precision computation to improve efficiency. All implementations were developed using PyTorch [26].

### Biological and genomic interpretation

To characterize factors associated with gene-level predictability from H&E morphology, we performed a regression analysis of spatial and molecular covariates, followed by an expression-matched pair analysis to isolate the specific impact of spatial organization from the confounding effect of transcript abundance.

#### Regression analysis of factors influencing gene expression predictability

To systematically characterize the factors governing gene expression predictability and to disentangle their independent effects, we formulated a multiple linear regression model encompassing 1,430 genes drawn from two biologically distinct domains: histopathological architecture genes (*n = 1,219*) and metabolic zonation genes (*n = 211*). The histopathological architecture domain consists of genes associated with tissue-level anatomical structures–such as vasculature, endothelium, and stromal compartments–as well as pathological features characteristic of primary sclerosing cholangitis (PSC), including inflammation, fibrosis, and collagen deposition. In contrast, the metabolic zonation domain captures the spatial stratification of liver metabolic functions, including cytochrome P450 enzymes and genes involved in lipid and glucose metabolism, which are known to exhibit well-defined lobular gradients [27]. By jointly modeling genes from these two domains, we aimed to assess how diverse biological and biophysical properties contribute to the predictability of spatial gene expression patterns.

To this end, we integrated multiple classes of explanatory variables into a unified regression framework, each representing a distinct aspect of spatial signal formation and observability, as described below.

***Spatial Pattern Strength*** We quantified the degree of spatial organization in gene expression using Moran’s I statistic as a standardized measure of spatial autocorrelation. For each tissue patch, a spatial weight matrix was constructed based on the six nearest neighboring patches defined by Euclidean distance in the tissue coordinate space. Higher Moran’s I values indicate genes with coherent spatial patterns–such as zonated bands or vessel-aligned expression–whereas lower values reflect diffuse or weakly structured spatial distributions. This metric captures the extent to which a gene’s expression exhibits structured spatial dependency beyond random variation.

*** Protein-Level Properties Related to Tissue Morphology*** The prediction targets in this study are RNA transcripts, but gene expression produces proteins that shape the cellular and tissue morphology captured by H&E staining. We therefore included subcellular localization, which indicates where within the cell the encoded protein resides, and protein isoelectric point (pI), which may influence staining contrast, as proxy variables to examine whether these protein-level properties correlate with gene-level predictability.

To evaluate whether subcellular compartmentalization constrains the emergence of observable spatial patterns, genes were grouped according to their annotated subcellular localization. Localization information was obtained from the Human Protein Atlas [28], and genes were categorized into six classes: nucleus, cytoplasm, mitochondria, membrane, extracellular matrix, and unknown or missing localization. This classification allowed us to test whether genes encoding proteins in broadly distributed compartments, such as the cytoplasm or extracellular matrix, are more predictable from H&E morphology than those confined to smaller compartments such as the nucleus.

To obtain pI values, protein sequences corresponding to each gene were retrieved from Ensembl using the biomaRt package [29], and pI values were computed with the Peptides R package based on the EMBOSS pK scale.

Finally, gene-level predictability, quantified using the gene-wise PCC defined in Section 2.3.3, was used as the dependent variable. Spatial autocorrelation (Moran’s I), subcellular localization, protein pI, and mean gene expression level (*M*) were included as independent variables.

#### Expression-matched analysis of spatial predictability

To isolate the independent impact of spatial pattern strength and mitigate potential confounding effects driven by transcript abundance, we implemented a 1:1 pair-matching protocol. Within each functional domain, successful genes with *PCC \ge 0.3* were paired with failed genes by identifying candidates that exhibited the minimum absolute difference in mean expression (*M*). This matching strategy was designed to neutralize abundance-driven bias, ensuring that the two groups were statistically comparable in terms of their transcript levels. Following the matching, we conducted a secondary statistical analysis on these controlled pairs to evaluate which variables retained significant influence over model performance when abundance was held constant. Furthermore, high-resolution spatial maps were generated for the matched pairs to enable a qualitative assessment of how distinct histological footprints and anatomical organization contribute to predictive success or failure. This dual approach allowed for a rigorous evaluation of the pure contribution of spatial and physical factors, independent of numerical transcript abundance.

## Results

**Table 1:** Performance comparison of single-patch gene expression prediction methods. The table reports PCCs for marker genes (MGs), highly expressed genes (HEGs), and highly variable genes (HVGs). Values are reported as mean PCC across three random seeds, with the maximum absolute deviation from the mean shown after ±. The baseline results for HisToGene, ST-Net, and BLEEP are cited from Xie et al. [17]. Bold values indicate the best results

Method (backbone)	MG (PCC)	HEG (PCC)	HVG (PCC)
HisToGene	*0.097 ± 0.015*	*0.072 ± 0.018*	*0.071 ± 0.011*
ST-Net	*0.099 ± 0.020*	*0.126 ± 0.005*	*0.091 ± 0.007*
BLEEP	*0.217 ± 0.002*	*0.175 ± 0.016*	*0.173 ± 0.011*
ResNet-18	*0.234 ± 0.010*	*0.271 ± 0.010 *	*0.215 ± 0.006 *
DenseNet-121	*0.265 ± 0.008 *	*0.295 ± 0.017*	*0.236 ± 0.002 *
EfficientNet-B7	$\mathbf {0.286 \pm 0.001}$	*0.305 ± 0.008*	* 0.239 ± 0.002*
EfficientNet-B0	*0.278 ± 0.009*	$\mathbf {0.310 \pm 0.005}$	$\mathbf {0.240 \pm 0.002}$

### Performance of the models

We benchmarked a range of pretrained convolutional models under identical training conditions to identify the most effective architecture for single-patch gene expression prediction. We evaluated ResNet-18, DenseNet-121, EfficientNet-B0, and EfficientNet-B7 and compared their performances with the established single-patch baselines: HisToGene, ST-Net, and BLEEP [17]. The proposed backbones were trained using the same dataset split and augmentation pipeline. To ensure a fair comparison, all backbones were evaluated using their optimal hyperparameters.

As summarized in Table 1, the EfficientNet architectures generally outperformed other backbones across all gene sets (MG, HEG, and HVG). Among all the evaluated models, EfficientNet-B0 achieved the highest performance in HEG and HVG sets, yielding mean PCC of 0.310 and 0.240. While EfficientNet-B7 achieved the highest correlation in the MG set with a PCC of 0.286, EfficientNet-B0 remained highly competitive with a PCC of 0.278. Notably, despite having the smallest number of parameters, EfficientNet-B0 surpassed other larger architectures in key metrics. These findings indicate that a lightweight convolutional backbone can effectively capture molecular information from the local histological morphology. EfficientNet-B0 thus demonstrated the highest predictive performance among the evaluated backbones, establishing a strong empirical baseline for future work on spatial gene expression prediction.

To strictly validate these performance differences, we performed further statistical analysis on the models trained in our controlled environment. We calculated the 95% confidence intervals based on the t-distribution for all evaluated backbones. This analysis confirmed the experimental stability of the reported performance. As illustrated in Supplementary Figure S1, the individual seed experiments for all backbones consistently fell within their respective estimated ranges. Furthermore, to statistically verify the pairwise superiority of the models, we conducted a paired Wilcoxon signed-rank test. This analysis was performed on a morphologically predictable gene set consisting of 49 genes, which are the union of genes predicted with a mean PCC of 0.3 or higher by at least one backbone. Our results show that EfficientNet-B0 significantly outperformed ResNet-18, DenseNet-121, and EfficientNet-B7. Detailed statistical comparisons are presented in Supplementary Table S1. Note that a PCC of 0.3 was adopted as a heuristic threshold of success and failure, representing the lower bound where predicted outputs begin to align with observable histological patterns, as illustrated in Supplementary Fig. S2. Informed by previous benchmarks such as BLEEP [17] and further supported by our quantitative analysis presented in Supplementary Table S1, this criterion serves as a flexible baseline that accounts for both statistical correlation and the visual presence of meaningful tissue-level patterns.

### Performance-efficiency comparison of models

For single-patch prediction, computational efficiency is as important as predictive performance. To quantify this trade-off, we analyzed how model size relates to predictive capability across different backbones (Table 2). Specifically, we compared the number of genes whose predicted expression achieved a PCC *\ge 0.3* against each model’s total parameter count.

Under identical training conditions, EfficientNet-B0 achieved the most favorable balance between accuracy and efficiency. It predicted 50 genes with a PCC *\ge 0.3* using only 5.3 M parameters, whereas BLEEP predicted only about 20 genes at this threshold [17]. In contrast, EfficientNet-B7 (66 M parameters) did not yield proportional performance gains, indicating diminishing returns with increasing model capacity. These findings suggest that a lightweight, parameter-efficient architecture can reliably capture informative morphological features to predict gene expression. In particular, EfficientNet-B0 demonstrated that high predictive power could be achieved without relying on large, resource-intensive backbones, reinforcing the suitability of compact CNN encoders for ST tasks.

**Table 2:** Performance-efficiency profile of different image encoders. The table compares the number of parameters against the number of reliably predicted genes (PCC *\ge 0.3*) on the test set, demonstrating backbone efficiency

Image encoder	# Parameters	# Genes (PCC *\ge 0.3*)
EfficientNet-B0	5.3 M	*50 ± 1*
DenseNet-121	8 M	*36 ± 5*
ResNet-18	11.7 M	*40 ± 3*
EfficientNet-B7	66 M	*41 ± 5*
BLEEP	26 M	*20 ± 1*

### Analysis of model configuration choices

To identify an efficient and stable model configuration, we systematically examined the effects of three critical design factors: (1) the data augmentation strategy, (2) backbone architecture and the number of hidden nodes (*U*), and (3) the number of hidden layers (*h*) and fine-tuning scope (*f*).

#### Comparison of data augmentation strategies

The MPA pipeline was compared with the augmentation protocol used in ST-Net [13]. As summarized in Table 3, the MPA approach consistently improved performance by reducing sensitivity to staining and imaging variability while preserving key morphological features. This improvement is particularly noticeable for ResNet-18 and DenseNet-121, suggesting that morphology-preserving transformations are more beneficial in models with weaker inductive biases. For the already robust EfficientNet-B0, the gain was modest but consistent across gene sets.

**Table 3:** Effect of data augmentation strategies on test set performance compared to the ST-Net protocol. Bold values indicate the best results

Backbone	Augmentation	MG (PCC)	HEG (PCC)	HVG (PCC)
ResNet-18	ST-Net	0.222	0.140	0.161
	MPA (Ours)	**0.234**	**0.271**	**0.215**
DenseNet-121	ST-Net	0.242	0.150	0.172
	MPA (Ours)	**0.265**	**0.295**	**0.230**
EfficientNet-B0	ST-Net	**0.278**	0.305	0.239
	MPA (Ours)	**0.278**	**0.310**	**0.240**
EfficientNet-B7	ST-Net	0.270	0.271	0.232
	MPA (Ours)	**0.286**	**0.305**	**0.239**

To further quantitatively validate the claim that MPA preserves tissue integrity better than aggressive transformations, we analyzed the consistency of feature embeddings before and after augmentation. We utilized a ResNet-18 encoder pretrained on 57 histopathology datasets [31], which is capable of capturing high-level histological features. We computed the cosine similarity between the feature vectors of original patches and those subjected to MPA versus aggressive augmentations, including cutout, grid distortion, and strong color jitter.

As presented in Table 4, MPA achieved a significantly higher mean cosine similarity of 0.928 compared to aggressive combined strategies at 0.851. While aggressive augmentations introduce greater variance, the lower similarity scores suggest a potential distortion of the underlying biological features. In contrast, the high feature conservation of MPA indicates that it successfully introduces necessary variations for training while maintaining the structural and semantic integrity of the tissue morphology.

**Table 4:** Quantitative analysis of structural integrity preservation. Feature consistency was measured using cosine similarity between original and augmented patches extracted by a domain-specific pretrained encoder [31]. Combined includes strong color jitter, grid distortion, and cutout. Bold values indicate the best results

Augmentation Method	Cosine Similarity (*↑ *)	Feature Consistency
**MPA (Ours)**	**0.928**	**High**
Color Jitter	0.907	Moderate
Grid Distortion	0.901	Moderate
Cutout	0.848	Low
Combined	0.851	Low

#### Architectures and number of hidden nodes

**Fig. 2:**
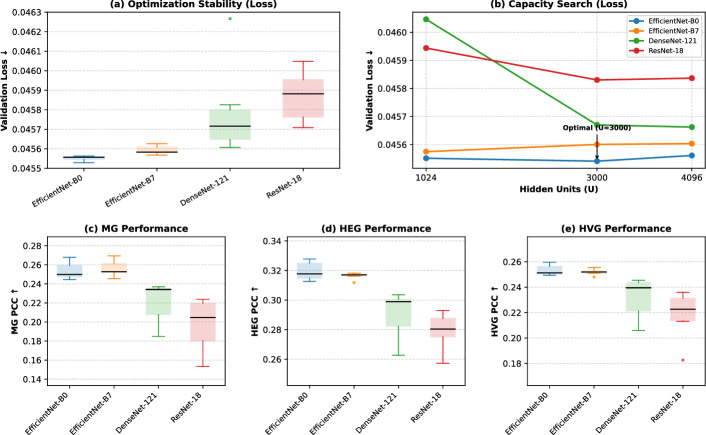
Architecture and capacity tuning results. All results are based on 5-fold cross-validation performance. **a** Distribution of mean validation loss for each backbone. Each backbone was evaluated across six configurations combining hidden layer depths $h \in \{1, 2\}$ and hidden unit sizes $U \in \{1024, 3000, 4096\}$. Each point represents the mean across folds for a specific configuration, and boxplots summarize the distribution across all six configurations. **b** Impact of hidden unit size *U* on mean validation loss, with values averaged over depths *h* for each backbone. **c**–**e** Distribution of mean PCC for MG, HEG, and HVG sets, respectively, across six configurations

Prior to fine-grained optimization, we conducted a broad search to select the most stable backbone and the optimal number of hidden nodes. We evaluated four representative backbone architectures and varying numbers of hidden nodes, $U \in \{1024, 3000, 4096\}$, under full fine-tuning. Figure 2 summarizes the stage 1 search results over the explored hyperparameter space, where each point reflects the mean validation performance from 5-fold cross-validation.

As shown in Fig. 2(a), the EfficientNet backbones show a tighter distribution of validation loss across the explored configurations, suggesting lower sensitivity to the hyperparameter choices than DenseNet-121 and ResNet-18. Regarding the number of hidden units (Fig. 2(b)), EfficientNet-B0 attains its lowest average validation loss at *U=3000*, while the differences across *U* are marginal and largely saturated at *U=4096*. Across gene sets, the aggregated PCC distributions (Fig. 2(c–e)) further indicate that the EfficientNet family consistently achieves top-tier correlations among the evaluated backbones. Therefore, we carried forward EfficientNet-B0/B7 to stage 2 and fixed *U=3000* as a standardized capacity setting, given the marginal loss differences across *U*.

#### Number of hidden layers and fine-tuning layers

**Fig. 3:**
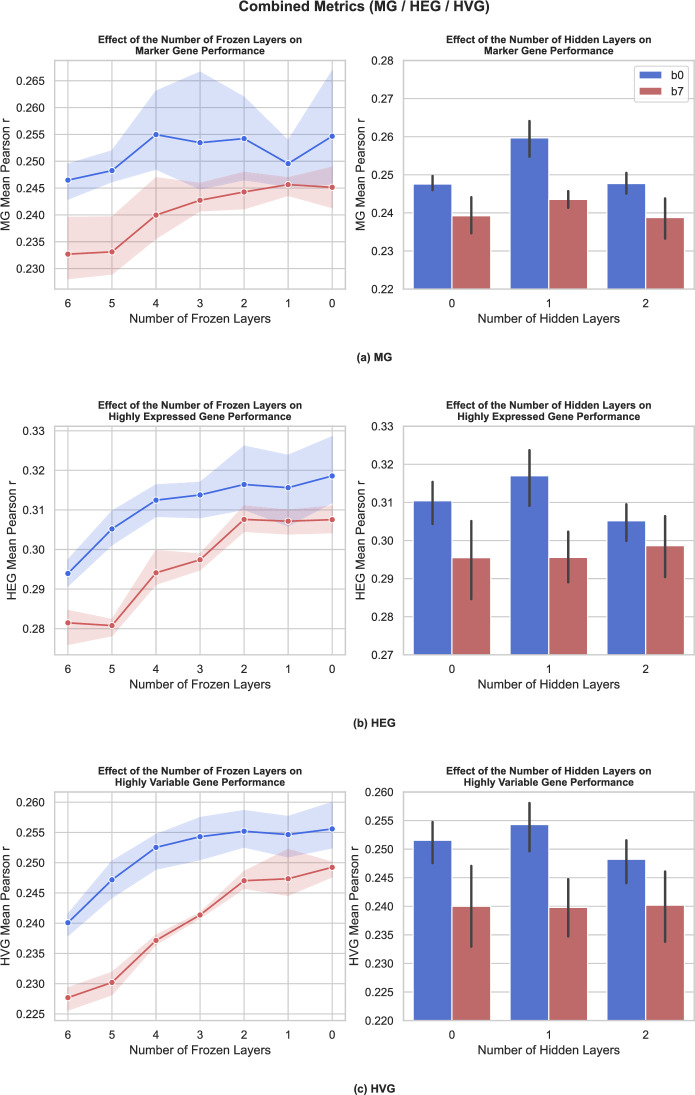
Optimization of backbone fine-tuning and network depth. Validation PCCs for EfficientNet-B0/B7 on MG, HEG, and HVG gene sets. Left: Backbone freezing impact averaged across MLP depths; shading shows variability. Right: MLP depth effect averaged across freeze levels; error bars show variability

Next, we performed a fine-grained optimization on the selected EfficientNet backbones to determine the optimal number of hidden layers *h* and fine-tuning scope *f*. As illustrated in Fig. 3, we compared linear baselines with *h=0* against non-linear heads with *h \ge 1* across the fine-tuning scope from $f \in \{0, \dots , 6\}$.

Introducing a single hidden layer consistently improved performance over the linear baseline, demonstrating the necessity of non-linear mapping. Performance peaked with full fine-tuning, indicating that adapting the entire backbone is essential for capturing domain-specific morphological features. Based on these optimization results, we determined that *U=3000*, *h=1*, and full fine-tuning at *f=0* achieved the best overall performance. Under this configuration, EfficientNet-B0 consistently outperformed other backbones and was therefore selected for downstream biological analyses.

### Biological and genomic interpretation

#### Global functional assessment via GO and KEGG

We first investigated whether the genes accurately predicted from single H&E patches represent biologically meaningful modules. To characterize the biological functions of predictable genes, we performed Kyoto Encyclopedia of Genes and Genomes (KEGG) and Gene Ontology (GO) enrichment analyses [32, 33] for the selected top 50 genes with PCC *\ge * 0.3.

**Fig. 4:**
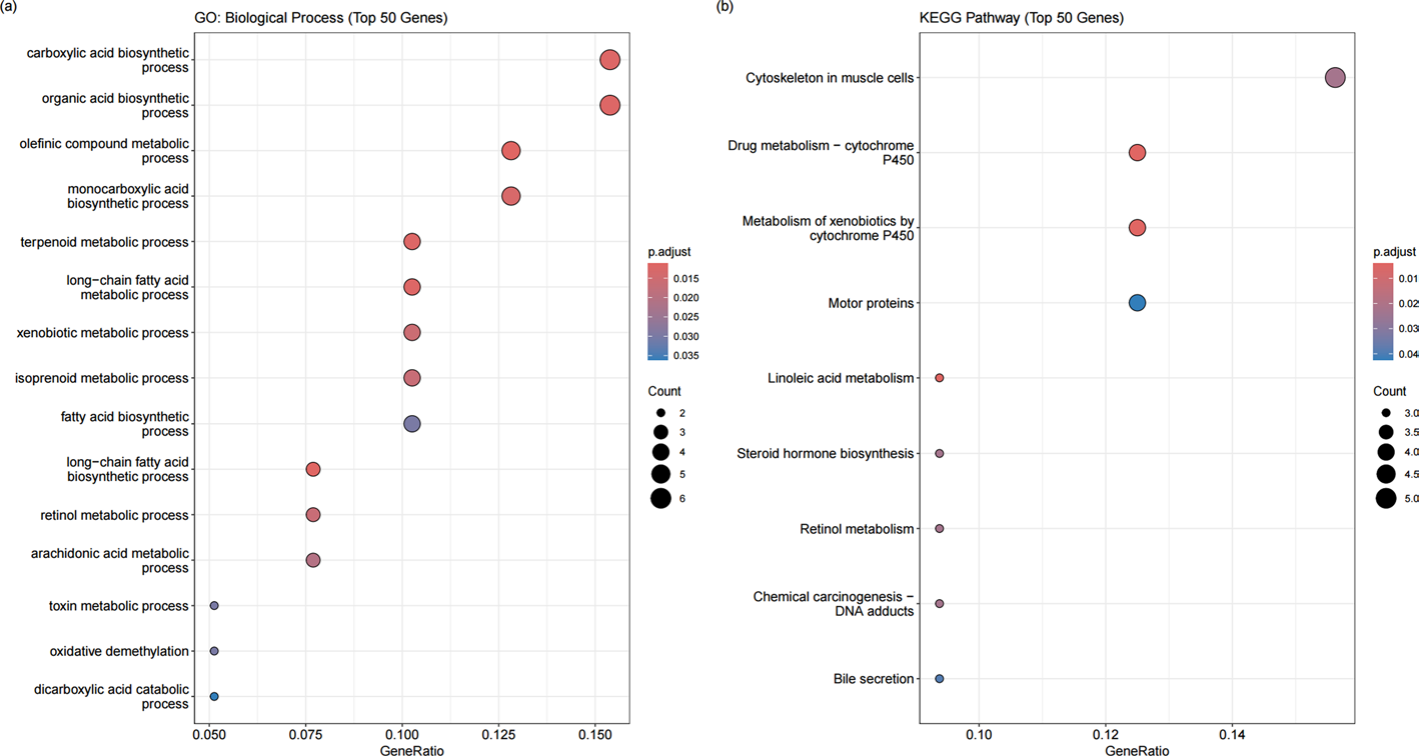
Functional enrichment analysis of the top 50 genes with PCC *\ge * 0.3. Left: GO Biological Process enrichment. Right: KEGG pathway enrichment. Dot size indicates the count of genes associated with each term, and the color gradient represents the adjusted *p*-value

Predictable genes showed significant enrichment in core hepatic programs, with the top pathways and their statistical details presented in Table 5 and Fig. 4. Drug metabolism pathways, including cytochrome P450 and xenobiotic metabolism, showed the strongest enrichment (*adj. p=0.0041*), with top genes including *CYP3A4* (PCC=0.75) and *CYP1A2* (PCC=0.70). The bile secretion pathway (*adj. p=0.0390*) reflected biliary stress characteristic of the PSC-affected tissue. Tissue remodeling genes such as *ACTA2* and *MYH11* were also recovered, demonstrating capture of both metabolic and structural signals. These results confirm that the model learns biologically meaningful hepatic programs rather than spurious image artifacts.

**Table 5:** Results of KEGG pathway enrichment analysis against highly predictable genes

Category	Pathway description	Adjusted *p*-value	Driving genes
Metabolism	Drug metabolism - cytochrome P450	0.0041	*CYP3A4*, *CYP1A2*, *CYP2E1*, *ADH6*
Metabolism	Metabolism of xenobiotics by P450	0.0041	*CYP3A4*, *CYP1A2*, *CYP2E1*, *ADH6*
PSC pathology	Bile secretion	0.0390	*CYP3A4*, *SLCO1B3*, *FXYD2*
Remodeling	Cytoskeleton in muscle cells	0.0226	*MYH11*, *TPM2*, *FBLN1*, *VIM*, *MYL9*
Remodeling	Motor proteins	0.0424	*MYH11*, *TPM2*, *MYL9*, *ACTA2*

#### Regression analysis of factors influencing gene expression predictability

This approach was designed to quantify the independent contribution of spatial structural strength while controlling for potential confounders such as transcript abundance within the biologically relevant functional domains. The constructed model achieved a robust explanatory power with an adjusted $R^2$ of 0.532 (*p < 2.2e-16*).

**Fig. 5:**
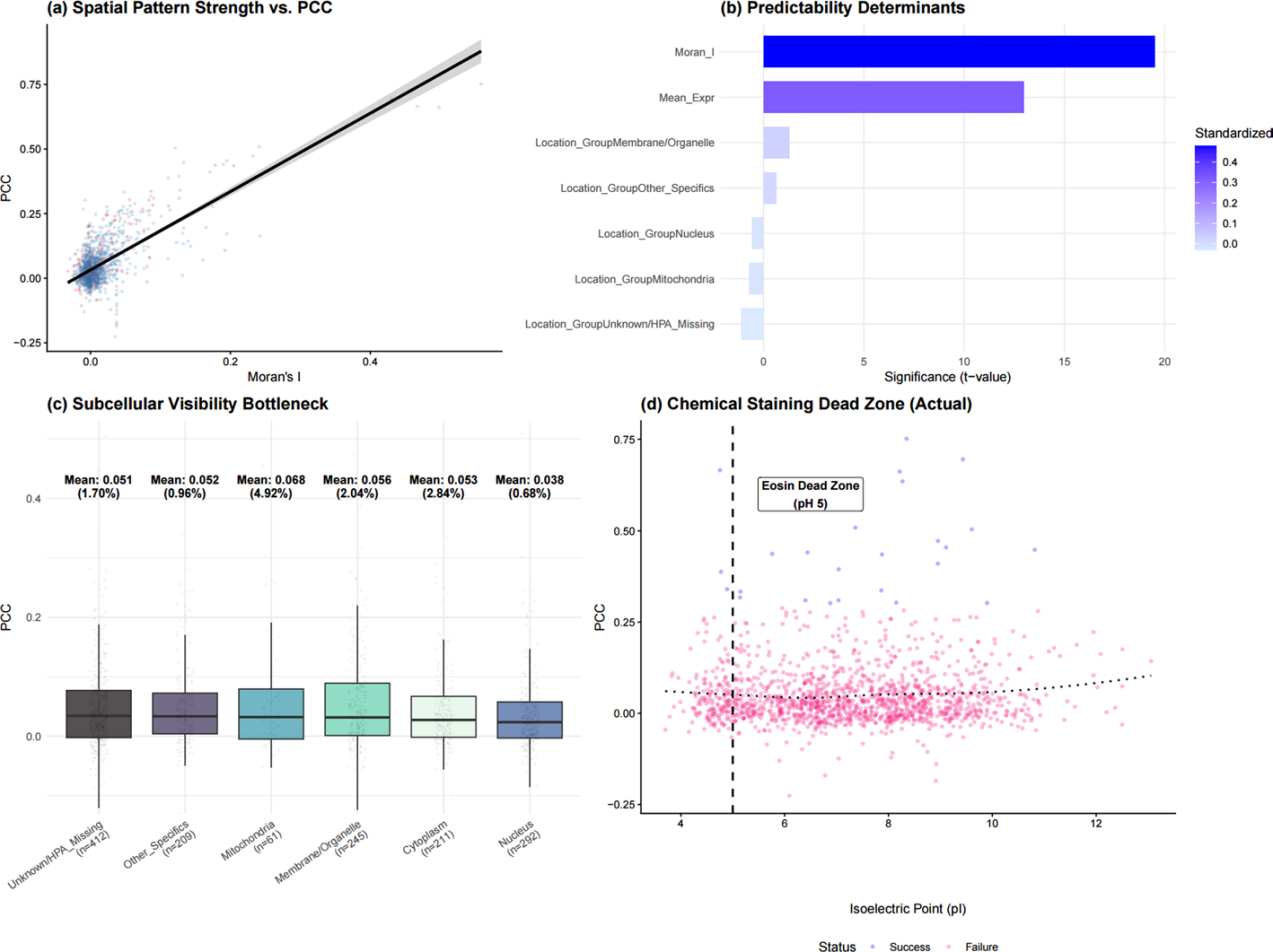
Global Determinants of Gene Expression Predictability. **A** Scatter plot of Moran’s I versus PCC. **B** Feature importance ranking of predictability variables. **C** Distribution of PCC across subcellular compartments. **D** Distribution of PCC against protein isoelectric points relative to the pH 5.0 reference line

The analysis identifies a clear hierarchy among the determinants, with spatial pattern strength acting as the primary driver of model success. As shown in Fig. 5A, Moran’s I exhibited a strong positive linear correlation with predictability (PCC). This dominance was statistically confirmed in the feature importance analysis (Fig. 5B), where Moran’s I recorded the highest significance level (*p < 0.001*) and a superior standardized coefficient (*β = 0.479*). While mean expression (*M*) also served as a significant prerequisite (*β = 0.318*), the inclusion of Moran’s I provided a substantial $R^2$ gain of 12.45% beyond abundance alone.

Beyond these primary drivers, the physical compartmentalization of proteins acts as a structural constraint on signal recovery. As shown in Fig. 5C, success rates varied by subcellular compartment, with mitochondria (*n=61*, *4.92%*) and cytoplasm (*n=211*, *2.84%*) showing higher predictability compared to the nucleus (*n=292*, *0.685%*). This suggests an observability limit where expansive cytoplasmic patterns are more effectively captured than nuclear-sequestered signals.

Finally, we evaluated chemical constraints related to pI. Even near pH 5.0, where a protein’s net charge approaches zero and reduces eosin staining contrast, no distinct performance collapse was observed (Fig. 5D). This indicates that pI-mediated contrast does not independently restrict predictability.

#### Expression-matched analysis of spatial predictability

**Fig. 6:**
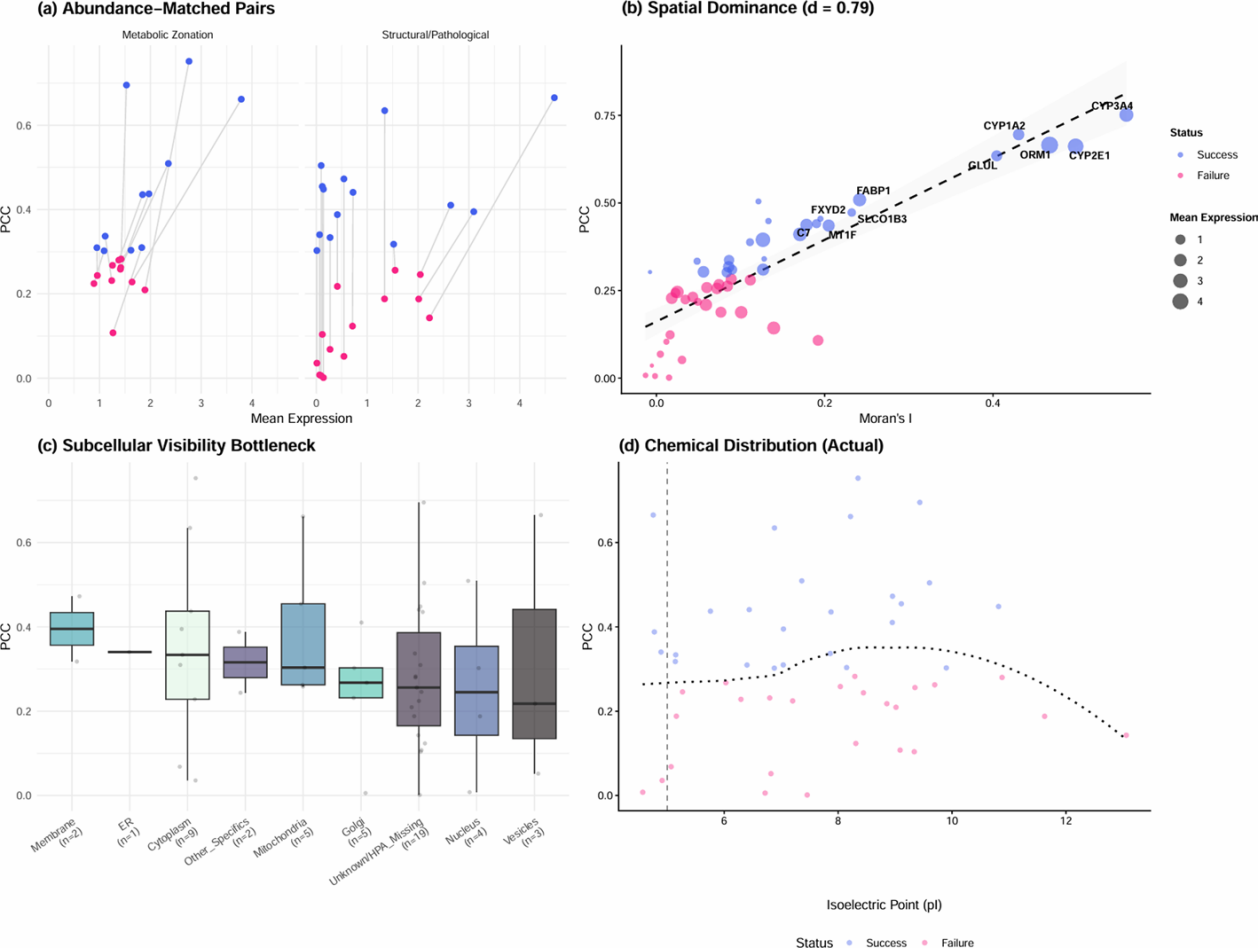
Analysis of Predictability Factors in Abundance-Matched Gene Pairs. **A** Comparison of mean expression (*M*) between success (PCC *\ge * 0.3) and failure (PCC < 0.3) groups across functional domains. **B** Relationship between spatial pattern strength (Moran’s I) and PCC for matched gene pairs. **C** Distribution of PCC across protein subcellular compartments for the matched cohort. **D** Scatter plot of PCC against protein isoelectric points (pI) relative to the pH 5.0 reference line

To disentangle the effect of spatial organization from confounding influences of transcript abundance, we performed a 1:1 expression-matched pair analysis. This procedure effectively eliminated abundance bias, as evidenced by the absence of a significant difference in mean expression between matched success and failure genes (Fig. 6A; *p = 0.439*).

Despite controlling for expression level, spatial pattern strength remained a strong determinant of predictive performance. Moran’s I exhibited a large effect size between success and failure groups within the matched cohort (Fig. 6B; Cohen’s *d = 0.79*), indicating that spatial autocorrelation exerts an independent influence on model accuracy.

The subcellular visibility constraint also persisted under abundance matching (Fig. 6C). In particular, genes localized to the cytoplasm continued to show a marked performance advantage, suggesting that physical compartmentalization imposes an intrinsic limit on recoverable spatial signals even when transcript levels are comparable. In contrast, chemical staining properties, approximated by pI, again showed no systematic association with prediction failure near the eosin dead zone (Fig. 6D).

Taken together, these results demonstrate that spatial organization and physical context, rather than transcript abundance or staining-related chemical properties, constitute the primary determinants of gene expression predictability from H&E morphology.

#### Spatial reconstruction and morphological concordance

**Fig. 7:**
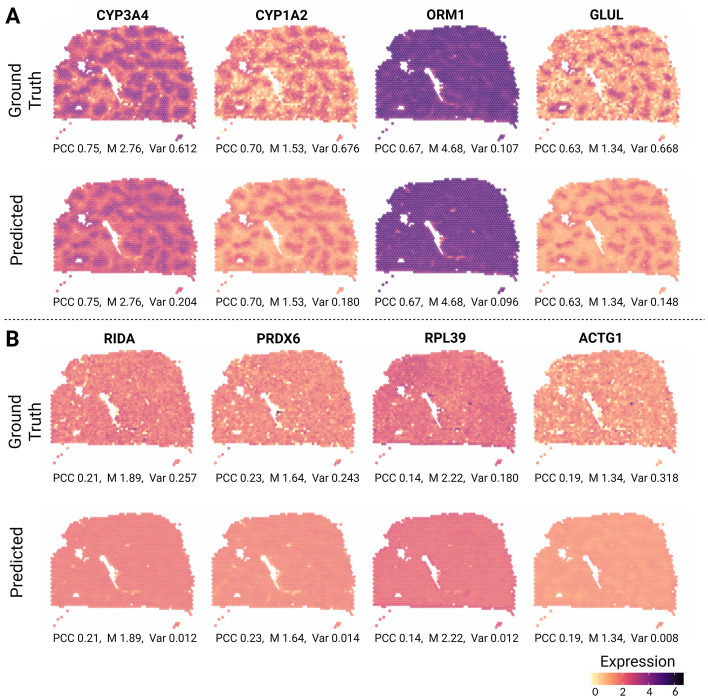
Spatial reconstruction of representative genes. **A** Genes with high predictive correlation (PCC *\ge * 0.3), including *CYP3A4*, *CYP1A2*, *ORM1*, and *GLUL*. **B** Genes with low predictive correlation (PCC < 0.3), including *RIDA*, *PRDX6*, *RPL39*, and *ACTG1*. For each gene, ground truth and predicted spatial expression maps are shown alongside their PCC, mean expression M, and variance Var

To examine whether predicted molecular outputs preserve anatomically meaningful spatial structure, we compared ground-truth and predicted expression maps for eight representative genes spanning metabolic zonation and histopathological architecture (Fig. 7). A complete list of the 50 genes evaluated in this analysis is provided in Supplementary File 1.

Clear qualitative differences emerged between successful and failed reconstructions. Genes shown in Fig. 7A, including *CYP3A4* (*PCC = 0.75*, *M = 2.76*), *CYP1A2* (*PCC = 0.70*, *M = 1.53*), *ORM1* (*PCC = 0.67*, *M = 4.68*), and *GLUL* (*PCC = 0.63*, *M = 1.34*), exhibited relatively well-defined spatial patterns and faithful reconstruction. These genes are characterized by either strong transcript abundance or distinct, anatomically anchored expression footprints. Notably, this observation aligns with prior landmark studies [27], which identify *GLUL* and *CYP2E1* as canonical pericentral markers forming robust lobular gradients aligned with vascular architecture.

In contrast, genes shown in Fig. 7B, including *RIDA*, *PRDX6*, *RPL39*, and *ACTG1*, produced diffuse and poorly localized predictions. Importantly, several of these genes displayed moderate to high transcript abundance–such as *RPL39* (*M = 2.22*) and *RIDA* (*M = 1.89*)–yet lacked stable, anatomically coherent expression patterns. The absence of such spatial anchoring prevents reliable reconstruction of tissue structure, reinforcing the conclusion that predictive success is governed by spatial organization rather than expression magnitude alone.

To further quantify this difference, we compared the predicted and ground truth variances for these representative genes. The high-PCC genes retained moderate predicted variances of 0.096–0.204 relative to their ground truth variances of 0.107–0.678, whereas the low-PCC genes exhibited near-complete mean collapse, where predicted values converge to a near-constant output, with predicted variances of 0.008–0.014 against ground truth variances of 0.180–0.318. While the model shows a general tendency toward variance reduction, this effect is less severe for genes with well-defined spatial expression patterns than for spatially unstructured genes.

### Cross-tissue validation on breast cancer dataset

To assess the generalizability of our single-patch baseline beyond the liver tissue, we performed external validation on a breast cancer dataset from [13]. To ensure a fair evaluation, we performed hyperparameter tuning on the breast cancer dataset by optimizing learning rate and weight decay via a slide-level grid search on one randomly selected patient (BC23803), and applied the selected configuration to all subsequent breast cancer experiments.

We first evaluated EfficientNet-B0 under slide-level splits within each patient. As shown in Table 6, EfficientNet-B0 achieved the best performance across all patients and gene sets, with the sole exception of MG in BC24105 where BLEEP achieved a higher score. However, overall prediction accuracy was lower than the liver benchmark (Table 1), suggesting that breast cancer tissue presents a more challenging prediction task. Performance also varied across patients, suggesting that prediction accuracy may differ across individual patients.

Under the patient-level split, EfficientNet-B0 achieved mean PCCs of 0.242 for MGs, 0.080 for HEGs, and 0.071 for HVGs on the independent test patient (Table 7). For comparison, ST-Net achieved PCCs of 0.214 for MGs, 0.069 for HEGs, and 0.064 for HVGs, while BLEEP achieved 0.182, 0.029, and 0.040, respectively. The statistical significance of performance differences between models was evaluated using Welch’s t-test, which indicated that EfficientNet-B0 achieved higher MG prediction than both ST-Net and BLEEP with *p<0.01*, although the absolute performance differences were modest. Furthermore, it consistently surpassed BLEEP across all gene categories at *p<0.01* and maintained performance comparable to ST-Net in the HEG and HVG sets with *p>0.05*, indicating that EfficientNet-B0 remains competitive across methods even under this stricter evaluation setting. Comparing the two evaluation settings, the patient-level split yielded lower performance than the slide-level split, particularly for HEGs and HVGs, suggesting that inter-patient heterogeneity poses an additional challenge.

**Table 6:** Slide-level split performance on the breast cancer dataset. Values are mean PCC ± standard deviation across three independent runs. Bold indicates the best performance for each gene set within each patient. Average row reports the mean ± standard deviation across patients

Patient ID	Method	MG (PCC)	HEG (PCC)	HVG (PCC)
BC23270	ST-Net	0.128 ± 0.010	0.078 ± 0.010	0.078 ± 0.010
	BLEEP	0.129 ± 0.010	0.083 ± 0.007	0.082 ± 0.007
	EfficientNet-B0	**0.146** ± 0.005	**0.101** ± 0.003	**0.100** ± 0.003
BC23803	ST-Net	0.105 ± 0.017	0.081 ± 0.023	0.078 ± 0.021
	BLEEP	0.112 ± 0.013	0.099 ± 0.009	0.097 ± 0.009
	EfficientNet-B0	**0.171** ± 0.004	**0.144** ± 0.003	**0.142** ± 0.004
BC24105	ST-Net	0.076 ± 0.019	0.052 ± 0.003	0.053 ± 0.004
	BLEEP	**0.163** ± 0.008	0.099 ± 0.022	0.100 ± 0.023
	EfficientNet-B0	0.142 ± 0.017	**0.114** ± 0.003	**0.115** ± 0.004
BC24220	ST-Net	0.255 ± 0.011	0.140 ± 0.004	0.137 ± 0.004
	BLEEP	0.288 ± 0.000	0.161 ± 0.000	0.156 ± 0.000
	EfficientNet-B0	**0.300** ± 0.010	**0.167** ± 0.004	**0.164** ± 0.004
Average	ST-Net	0.141 ± 0.068	0.088 ± 0.032	0.086 ± 0.031
	BLEEP	0.173 ± 0.069	0.111 ± 0.030	0.109 ± 0.028
	EfficientNet-B0	**0.190** ± 0.065	**0.132** ± 0.026	**0.130** ± 0.025

**Table 7:** Cross-tissue validation performance on breast cancer dataset. Values are mean PCC ± standard deviation across five independent runs. Bold indicates the best performance, and underline indicates the second-best performance for each gene set. ** denotes statistical significance (*p < 0.01*) compared to both baseline methods

Method	MG (PCC)	HEG (PCC)	HVG (PCC)
ST-Net	$\underline{0.214 \pm 0.014}$	$\underline{0.069 \pm 0.010}$	$\underline{0.064 \pm 0.011}$
BLEEP	*0.182 ± 0.027*	*0.029 ± 0.004*	*0.040 ± 0.004*
**EfficientNet-B0**	$$\mathbf {0.242 \pm 0.006}^{**}$$	$$\mathbf {0.080 \pm 0.009}$$	$$\mathbf {0.071 \pm 0.007}$$

## Discussion

Our systematic benchmarking identified the EfficientNet-B0 backbone as the most effective architecture for predicting single-patch spatial gene expression. Under identical training conditions, the EfficientNet models consistently outperformed previous single-patch approaches, including HisToGene, ST-Net, and BLEEP, across all gene sets. Among all the evaluated backbones, EfficientNet-B0 achieved the best overall performance while maintaining the smallest number of parameters, demonstrating that high predictive accuracy does not require large-scale or Transformer-based architectures. This finding underscores the importance of architectural efficiency when modeling histopathological images, where morphological cues can be effectively captured by compact convolutional networks.

The robustness of the model was further supported using an MPA pipeline. Compared to the augmentation protocol from ST-Net, MPA consistently yielded higher predictive correlations, indicating that proper geometric perturbations can help identify histological patterns for molecular inference. These results highlight the importance of data augmentation strategies for preserving biologically meaningful tissue morphologies. Moreover, the fine-tuning depth also plays a decisive role in performance. Systematic evaluation revealed that full fine-tuning of the backbone consistently provided the highest accuracy across gene sets, whereas partial freezing of layers limited the ability of the model to adapt to domain-specific morphological features. This suggests that ImageNet-pretrained features, which are valuable for initialization, require complete adaptation to effectively capture the complex texture and organization of biological tissues.

The biological interpretation of our results indicates that the model learns biologically meaningful hepatic programs rather than mere image artifacts. KEGG enrichment analysis identified core functional modules, including drug metabolism, bile secretion, and cytoskeletal organization, demonstrating that the model can simultaneously capture parenchymal metabolic activity and stromal or architectural reactions from local histological cues.

Moreover, our abundance-controlled pair-matching analysis confirmed that spatial organization is the primary driver of predictive accuracy. Even at comparable expression levels, genes with high Moran’s I achieved substantially higher predictability than spatially diffuse genes, as illustrated by *CYP3A4* versus *RIDA*. Transcript abundance was necessary but not sufficient on its own, as genes lacking structured spatial patterns remained difficult to predict regardless of their expression levels. These results suggest that the observed performance ceiling reflects an intrinsic limit in the spatially organized information available in H&E histology, rather than a shortcoming addressable by more data or larger models alone. Neither protein isoelectric point nor subcellular localization showed a dominant independent effect, though these protein-level proxies should be interpreted with caution given their indirect relationship to RNA-based predictability.

As this benchmark is based on four sequential sections from a single healthy human liver, it does not assess cross-donor generalization. The breast cancer experiments were designed to complement this limitation by evaluating the framework across different tissue types and patients. The observed performance gap between liver and breast cancer tissue, even under equivalent slide-level split conditions, suggests that prediction difficulty is tissue-dependent, potentially reflecting differences in the degree of spatial organization, such as the well-characterized lobular zonation of normal liver [27]. The further performance drop under the patient-level split highlights that inter-patient variability remains a substantial challenge for morphology-based gene expression prediction. These observations point to tissue-type characterization and patient-level robustness as important considerations for future spatial transcriptomics models.

A potential issue is the tendency of regression-based predictors to converge toward mean expression, as identified by Xie et al. [17] in models such as HisToGene and ST-Net. As EfficientNet-B0 is trained with MSE loss, it is also susceptible to this phenomenon. From the representative genes examined in Fig. 7, we confirmed that predicted variance was consistently lower than ground truth variance, indicating that a certain level of mean collapse is present. Notably, this reduction tended to be less pronounced for genes with higher ground truth variance, suggesting that mean collapse is less severe for genes with well-defined spatial expression patterns. However, this observation is based on a limited set of representative genes and has not been quantitatively validated across the full gene set. A comprehensive characterization of mean collapse would be a valuable direction for future research, particularly when evaluating models that incorporate spatial context, where it is important to quantify how much mean collapse is mitigated relative to single-patch baselines.

Accordingly, future progress will require strategies that explicitly expand the amount of spatially organized information available to the model. Multimodal integration approaches that incorporate complementary sources of spatial context may provide additional structural cues beyond those present in standard H&E morphology, thereby enabling more reliable inference for genes that currently lack discernible histological footprints. Consistent with this perspective, recent multimodal frameworks such as GCNVDA [34] and VIMCCA [35] have demonstrated that integrating functional gene modules and heterogeneous data sources is critical for capturing biological variability that cannot be resolved within a single modality.

## Conclusion

We conducted a systematic evaluation of multiple backbone architectures and training strategies for single-patch spatial gene expression prediction on liver tissue, with additional validation on breast cancer tissue to assess cross-tissue generalizability. EfficientNet-B0, when fully fine-tuned and trained with Morphology-Preserving Augmentation (MPA), achieved the best balance between predictive accuracy and computational efficiency. This study provides a computationally efficient and reproducible reference for the field by defining a lightweight yet high-performance baseline for single-patch inference. Through subsequent biological and genomic interpretations, we further demonstrate that predictive performance is fundamentally constrained by the amount of spatially organized information physically encoded in H&E histology. Genes that form coherent tissue-level patterns can be reliably inferred from local morphology, whereas genes lacking such structured spatial organization remain difficult to predict, even when transcript abundance is sufficient. In contrast, factors such as protein isoelectric point or subcellular localization exhibited only minor or non-dominant effects relative to spatial pattern strength and did not emerge as independent drivers of predictability. Together, these findings indicate that the primary limitation arises from intrinsic information boundaries of morphology-based inference rather than from model capacity or training data volume. Importantly, by restricting the model to single-patch inputs, this study enables systematic interrogation of which biologically interpretable factors are sufficient–or insufficient–for morphology-based gene expression prediction. By explicitly defining this baseline and its governing factors, this study provides a principled reference for evaluating how additional spatial context, multiresolution designs, or multimodal integration can extend beyond the limits of single-patch morphology-based gene expression prediction.

## Supplementary Information

Below is the link to the electronic supplementary material.

Supplementary file (csv 7 kb)

## Data Availability

The human liver Visium dataset is publicly available in the NCBI Gene Expression Omnibus (GEO) under accession number GSE240429 (Andrews et al., 2024). The breast cancer spatial transcriptomics dataset is from the ST-Net study (He et al., 2020; Stenbeck et al., 2021), accessible through the original publication. The source code for this study is openly available at the following URL: https://github.com/KU-MedAI/MAI-spatial-transcriptomics

## Abbreviations

GO: Gene Ontology
GEO: Gene expression omnibus
H&E: Hematoxylin and eosin
HEGs: Highly expressed genes
HVGs: Highly variable genes
KEGG: Kyoto Encyclopedia of Genes and Genomes
MGs: Marker genes
MPA: Morphology-preserving augmentation
PSC: Primary sclerosing cholangitis
ST: Spatial transcriptomics
WSI: Whole-slide image
